# The Efficacy of Wireless Auditory Training in Unilateral Hearing Loss Rehabilitation

**DOI:** 10.3390/audiolres14040046

**Published:** 2024-06-24

**Authors:** Andrea Lovato, Daniele Monzani, Ylenia Kambo, Leonardo Franz, Andrea Frosolini, Cosimo De Filippis

**Affiliations:** 1 Otorhinolaryngology Unit, Department of Surgical Specialties, Vicenza Civil Hospital, 36100 Vicenza, Italy; kamboylenia@gmail.com; 2 Otorhinolaryngology Unit, Department of Surgical Specialties, University of Verona, 37100 Verona, Italy; daniele.monzani@univr.it; 3 Audiology Unit at Treviso Hospital, Department of Neuroscience DNS, University of Padova, 31100 Treviso, Italycosimo.defilippis@unipd.it (C.D.F.); 4 Maxillofacial Surgery Unit, Department of Medical Biotechnologies, University of Siena, 53100 Siena, Italy

**Keywords:** auditory training, unilateral hearing loss, wireless, hearing rehabilitation, binaural hearing

## Abstract

Purpose: The purpose of this study was to evaluate the efficacy of auditory training (AT) in patients with unilateral hearing loss (UHL) using hearing aids (HAs), comparing traditional methods with a new approach involving a wireless remote microphone. Methods: The study included 96 participants, divided into two groups, with ages ranging from 42 to 64 years, comprising both male and female subjects. A clinical trial including consecutive moderate UHL patients was performed at our institution. For the study group, a Roger Pen was used during AT with patients inside a sound-attenuating cabin. Controls followed conventional sessions. Professional speech and language pathologists performed the rehabilitation. Audiological outcomes were measured, including word recognition at signal-to-noise ratios (SNRs) of 0 dB, +5 dB, and +10 dB, to determine the effectiveness of the training. Measurements also included the Speech, Spatial, and Qualities of Hearing Scale to assess perceived auditory abilities. Results: A total of 46 and 50 UHL patients were randomly included in the study and control groups, respectively. No differences were found in terms of sex, age, presence of tinnitus, duration of hearing loss, pure tone average, and speech-in-noise perception without an HA. Following HA fitting and AT, a notable enhancement in the ability to identify speech in noisy environments was observed in the study group. This improvement was significant at SNRs of +5 and +10. When comparing the ability to identify speech in noise using HAs across both groups, it was observed that hearing capabilities post-wireless AT showed a significant improvement at an SNR of +5. Only the study group had a significant improvement in the total Speech, Spatial, and Qualities of Hearing Scale score after the training. Conclusions: In our group of UHL patients, we found significantly better speech-in-noise perception when HA fitting was followed by wireless AT. Wireless AT may facilitate usage of HAs, leading to binaural hearing in UHL patients. Our findings suggest that future interventions might benefit from incorporating wireless technology in AT programs.

## 1. Introduction

While hearing aids are the gold standard to treat hearing loss, their users often encounter significant challenges in complex everyday situations, like understanding speech amid background noise [1]. Auditory training (AT) has a pivotal role in aural rehabilitation together with an adequate prosthetic prescription (i.e., hearing aids and cochlear implants), audiological fitting, patient instruction, and counseling [2]. Mitigating the functional, activity-related, participatory, and quality-of-life impairments resulting from hearing loss is the objective of AT through listening exercises and rehabilitation [3]. Historically, auditory training (AT) is conducted through direct, in-person sessions led by a speech and language expert. Nevertheless, various alternatives have emerged, including group-based sessions or personalized training at home [4]. In 2013, Henshaw and colleagues presented a comprehensive review evaluating the advantages of computer-assisted AT for adults with hearing impairments. This review found solid evidence of enhanced performance in trained auditory tasks [5]. AT could be performed either with conventional in-person sessions or with computer-assisted technologies, and its benefits could apply to hearing aids (HAs) or cochlear implant users. Many authors reported the positive role of AT in HA usage [6,7,8]. One earlier study investigated the impact of phoneme discrimination training for hearing aid users with mild-to-moderate hearing loss [9]. The findings indicated notable improvements post-training in a listening task involving competing speech, with an improvement of 2.3 dB in signal-to-noise ratio (SNR) [9]. Anderson et al. (2013) studied how auditory-based cognitive training affects the precision of subcortical speech processing in noise [6]. Following the training, older participants showed quicker neural responses and reported improvements in memory, processing speed, and perception of speech in noise, in contrast to a control group that exhibited no change [6]. Conversely, a randomized controlled trial involving 279 veterans compared outcomes of standard hearing aid (HA) intervention with various AT methods combined with HAs [8]. The study, using performance and self-report metrics, concluded that AT did not enhance outcomes beyond what was achieved with standard HA intervention alone [8].

In unilateral hearing loss (UHL) patients, HA fitting needs to take into account the effect of normal hearing on the contralateral side [10,11]. In a comprehensive analysis conducted in South Korea focusing on UHL and the use of hearing aids (HAs), researchers discovered that the decision to adopt HAs was influenced by the hearing threshold levels in both the better and worse ear [12]. The role of AT in UHL aural rehabilitation has been scarcely investigated in the literature, even though a recent preliminary report supports the effectiveness of specifically designed rehabilitation methods for this population of patients [13]. Phonak’s Roger Pen is an advanced remote microphone system featuring digital adaptive multi-channel technology, which wirelessly channels the speaker’s voice directly to the sound processor of an HA [14]. The use of remote wireless microphones has demonstrated considerable enhancements in speech perception within noisy environments in quasi-experimental studies [15]. However, the implementation of these technologies in repeated AT sessions has not been explored, to the best of our knowledge.

In this study, we conducted an RCT to test the hypothesis that wireless direct transmission to an HA during in-clinic AT could improve the audiological outcome for UHL patients.

## 2. Materials and Methods

### 2.1. Participants

The study included 96 participants, divided into two groups, with ages ranging from 42 to 64 years, comprising both male and female subjects. The study focused on consecutive patients with UHL who were potential candidates for using an HA. Inclusion criteria were (i) the presence of unilateral moderate hearing loss, characterized by a pure tone average (PTA) ranging from 41 to 70 dB HL; and (ii) a PTA (average of hearing threshold levels at 500, 1000, 2000, and 4000 Hz) of less than 15 dB in the contralateral ear. Exclusion criteria were (i) psychiatric and/or neurological comorbidity; (ii) taking medications that interfere with normal cognitive functioning (for example, anxiolytics, antiepileptics, antidepressants). This randomized clinical trial was carried out in line with the Helsinki Declaration guidelines and received approval from our internal Ethics Committee (reference no. 01-2019AU). Data handling complied with Italian privacy and sensitive data legislation. Every participant in the study provided their agreement by signing a comprehensive informed consent form. Demographic and clinical data such as sex, age, etiology of hearing loss, and duration of deafness were recorded. After receiving comprehensive audiological evaluation (see Section 2.2), all patients were fitted with unilateral HAs (see Section 2.3) and were randomized into two groups using Excel RAND function (Microsoft, Washington, DC, USA). The study group underwent AT (see Section 2.4) using a Roger Pen (Sonova, Stäfa, Switzerland) while the control group were treated with conventional AT (i.e., direct in-person sessions led by a speech and language expert without using wireless technology). Randomization was not blinded and patients knew what treatments were performed. Patients were re-evaluated after two months (i.e., one month for HA acclimation, and one month for AT) with the audiological test battery herein described.

### 2.2. Audiological Evaluation

Audiometric assessments were conducted using the Madsen Astera by GN Otometrics (Ballerup, Denmark), adhering to the European (IEC 60645-I) [16] and ISO (389-1) standards [17], in a soundproof room. Hearing thresholds were evaluated across frequencies from 250 to 8000 Hz. When assessing the ear with UHL, masking was applied to the contralateral ear, as previously detailed [14,18]. Hearing loss severity was determined by the PTA across 0.5, 1, 2, and 4 kHz, categorized as mild (PTA 21–40 dB HL), moderate (PTA 41–70 dB HL), severe (PTA 71–95 dB HL), or profound (PTA > 95 dB HL), following standard classification [19]. Speech perception and binaural hearing capabilities were assessed using a speech-in-noise perception test. This test utilized disyllabic, phonetically balanced words from an Italian wordlist [20]. The speech-in-noise test included 25 words at 65 dB, presented with white noise. The accuracy of word recognition was recorded at signal-to-noise ratios (SNRs) of 0 dB, +5 dB, and +10 dB, with both signal and noise emanating from the front. Additionally, all participants completed the Speech, Spatial, and Qualities of Hearing Scale (SSQ) [21], a 49-item questionnaire that evaluates perceived hearing abilities in various scenarios using a Likert scale.

### 2.3. Hearing Aid

All patients were fitted with a Phonak Bolero B70-M (Sonova, Stäfa, Switzerland) in order to assure connectivity with the Roger Pen in the study group and avoid bias with the control group. For the fitting, we used a curvilinear compression input/output hearing aid formula, which is a frequency-specific mathematical approach that describes the relationship between the input level of a signal delivered to the hearing aid and the output level produced by the hearing aid. Acclimation to the hearing aid was achieved in four weeks with progressive augmentation of HA stimulation (during four consecutive regulations). AT was started after these four weeks.

### 2.4. Auditory Training

In our department, AT is the standard of care and is conducted by a speech and language pathologist (SLP) through direct, in-person sessions, incorporating both bottom-up (analytic) and top-down (synthetic) exercises [22]. The analytic method focuses on context-independent acoustic–phonetic signals, training individuals to interpret speech signals devoid of any contextual clues, like syllabic patterns, vowel sounds, and differences in initial consonants. Conversely, the synthetic method leverages the listener’s linguistic understanding (including semantic, syntactic, lexical, and phonological knowledge) to compensate for the incomplete sensory data received through their hearing device.

Specifically, during AT the following therapeutic interventions were used: Auditory Discrimination Tasks: These tasks involve identifying and discriminating between auditory stimuli based on specific auditory features, such as pitch, duration, intensity, and frequency. Activities may include sorting sounds, identifying patterns, and recognizing changes in sound sequences.

Auditory Closure Exercises: Auditory closure exercises involve completing or filling in missing parts of auditory stimuli. For example, individuals may listen to incomplete words or sentences and attempt to predict or fill in the missing sounds based on context and auditory cues.

Auditory Memory Training: Auditory memory training focuses on improving the individual’s ability to retain and recall auditory information over time. Activities may include repeating sequences of sounds or words, recalling auditory stimuli presented earlier, and practicing auditory recall in challenging listening conditions.

Auditory Sequencing Activities: These activities involve arranging auditory stimuli in a specific order based on auditory cues. For example, individuals may practice sequencing sounds, syllables, or words to form meaningful patterns or sequences.

Auditory Feedback and Monitoring: Throughout AT, the SLP provides real-time feedback and monitoring to help individuals track their progress, identify areas for improvement, and adjust their listening strategies accordingly. This feedback fosters self-awareness and self-correction, empowering individuals to become active participants in their auditory rehabilitation.

Herein, we report some examples of auditory tasks:

i. Phonemic Contrasts: AT involves practicing discriminating between phonemes that are acoustically similar but linguistically distinct. For example, distinguishing between /s/ and /ʃ/ or /p/ and /b/ helps sharpen phonemic awareness and improve speech perception.
ii. Minimal Pairs: Minimal pairs are pairs of words that differ by only one phoneme, such as “cat” and “bat” or “ship” and “sheep.” By contrasting these minimal pairs, individuals with hearing loss can refine their ability to detect subtle differences in speech sounds.
iii. Syllable and Word Repetition: Repetition of syllables and words with varying phonetic features helps reinforce auditory discrimination skills and improve auditory memory. This may involve repeating monosyllabic words (e.g., “bat,” “cat”) or multisyllabic words (e.g., “elephant,” “banana”).
iv. Sentences and Connected Speech: AT progresses to more complex linguistic units, such as sentences and connected speech. Individuals practice listening to and comprehending sentences of increasing length and complexity, as well as conversational speech, to improve their ability to extract meaning from context.

For the study group, the training was started post-HA fitting and consisted of 60 min sessions twice a week for one month. Before starting AT, the Roger Pen was coupled to the HA recipient via the appropriate receiver. To ensure efficient isolation of the contralateral normal ear, we used a combined solution: the patient was located in a soundproof booth while wearing an individual customized ear mold. All AT exercises were performed by the speech therapist using a wireless microphone in a body-worn setting, without patient access to lip reading, but visually monitored through the soundproof booth window. For control group patients, AT was performed with conventional 60 min face-to-face sessions twice a week for the same 1-month period. After the one-month training, the groups were re-assessed using the same evaluative tools as those employed before the training commenced.

### 2.5. Statistics

In order to determine the minimum number of subjects to enroll in a study for adequate power, we considered an expected effect size of 2.8 dB, a standard deviation (SD) of the PTA of 6 dB, a power of 80%, and a significance of 0.05. The expected effect size was informed by previous data and expert consultation [23], representing the minimum clinically significant difference we aimed to detect between the intervention and control groups. The standard deviation was based on historical data from similar patient populations [24]. Utilizing these parameters, we employed the standard formula for comparing two means in a two-sided test, accounting for equal variance and sample size in both groups. This calculation resulted in a sample size of 45 participants per group, which was rounded up to the nearest whole number to account for potential dropouts and ensure adequate power. Our sample size determination was integral to the study’s design, ensuring that we could reliably detect a meaningful difference in outcomes between the study groups. For our statistical analyses, we employed the Mann–Whitney U test, the chi-square test, and the Wilcoxon signed-rank test, depending on the suitability for each data set. We chose non-parametric tests which could be more reliable when dealing with small sample sizes, where the assumptions of parametric tests may not hold. We established statistical significance at a *p*-value of less than 0.05. All data analysis was conducted using SPSS version 17 for the Social Sciences (SPSS Inc., Chicago, IL, USA).

## 3. Results

We enrolled 46 consecutive patients with UHL in the study group (comprising 20 females and 26 males, with an average age of 56.7 years), who underwent wireless AT following HA fitting. The control group included 50 participants who received traditional AT sessions (26 females and 24 males, average age of 58.2 years). The etiology of hearing loss is detailed in Table 1. We assessed and compared the demographic and clinical characteristics of both groups prior to HA fitting, and the findings are presented in Table 2: there were no significant differences between the two groups.

Table 3 presents the outcomes of the speech-in-noise test with an HA following the training. In the study group, a marked improvement was noted at SNRs of +5 (*p* = 0.002; Wilcoxon signed-rank test) and + 10 (*p* = 0.02; Wilcoxon signed-rank test), while the control group showed no significant improvements. When comparing the speech-in-noise results of the two groups, it was observed that hearing performance post-wireless AT was significantly better at an SNR of + 5 (*p* = 0.041; Mann–Whitney U test), but no notable differences were evident at SNRs of 0 and + 10 (*p* = 0.60 and *p* = 0.08, respectively; Mann–Whitney U test). Only the study group demonstrated a significant enhancement in the total SSQ scores after training (increasing from a mean of 4.1 to 7.3, *p* = 0.03, compared to the control group’s increase from a mean of 4.8 to 7.0, *p* = 0.7; Mann–Whitney U test).

## 4. Discussion

Prior research on AT has predominantly focused on bilateral hearing loss, with UHL patients being less frequently the subject of study. Previous studies confirmed a positive role of AT in patients with bilateral hearing loss: AT facilitates acclimation to hearing aids in bilateral hearing loss [6,7,9]. In UHL patients, acclimation to HAs is considered difficult as the normal ear is dominant. We described a similar effect in asymmetric hearing loss: these patients chose HAs more frequently in the best hearing side [10]. Conventional AT stimulates both ears at the same time; consequently, it may be less efficient in UHL patients. In the present study, we evaluated AT using wireless technology in order to better stimulate the affected ear.

We investigated the audiological outcome of consecutive UHL patients that underwent AT after unilateral HA fitting. Patients were randomized into two groups in order to evaluate whether the use of wireless technology (Roger Pen) during AT could improve the outcome of unilateral HA use. The Roger Pen operates as a directional microphone. Positioned close to the speaker’s mouth, it captures auditory stimuli which are then transduced into an electrical signal that undergoes wireless transmission directly to the auditory processing unit of the HA. This method of capturing the signal at or near its source effectively minimizes the adverse impacts of background noise and distance, thereby enhancing the SNR at the listener’s ear [14]. In the study group, we used the Roger Pen, which directly stimulated the ear with an HA during AT. Furthermore, during the rehabilitation the patients were inside a sound-attenuating cabin, so the normal ear was isolated. This method was applied uniformly in the study group. Only the remote microphone used by the speech therapist outside the cabin stimulated the HA. In the study group, a significant improvement was observed in the speech-in-noise tests at SNRs of + 5 and + 10, while in the control group no significant changes were detected. There is a lack of previous studies focusing on UHL patients in this context. The assessment of the advantages of binaural hearing in UHL patients remains a topic of ongoing debate. Previous researchers have reported the difficulties of capturing significant enhancements in audiological tests following device fitting in patients with UHL [25]. For instance, in patients with conductive UHL due to congenital aural atresia, Danhauer et al. (2010) evaluated the impact of BAHA implant systems compared to unaided conditions. Although audiometric evaluations revealed minimal differences with and without BAHAs, the majority of subjects perceived an improvement in their quality of life with BAHAs [26]. Similarly, in cases of sensorineural UHL patients using conventional HAs, while speech perception scores did not show a significant benefit when aided, subjective assessments indicated a substantial improvement in quality of life [27]. In our study, we found significant improvements in total SSQ scores and at audiological examinations only in patients treated with wireless AT. We chose the SSQ questionnaire as it has been frequently used in assessing UHL patients [28,29]. According to our results, wireless AT facilitated and accelerated HA habituation with better binaural hearing. Auditory training stimulated neuroplasticity and perceptual learning in bilateral hearing loss patients using HAs [1]. We can hypothesize a similar process in UHL patients. In our study, the follow-up was short, and we would need a longer observational period to evaluate neuroplasticity in UHL patients.

## 5. Study Limitations

The primary limitations of this study were its single-center design and the absence of a comparison with an untreated group. Other limitations were the limited number of included patients and use of only bisyllabic words in the auditory tests. The follow-up was short. Nevertheless, we started the one-month AT after four weeks of HA acclimation; consequently, the follow-up time was two months. A key strength of this study is the uniformity of the two groups examined, as well as the adoption of both objective and subjective standard audiological evaluation methods. In the future, patients with UHL or asymmetrical hearing loss could benefit from AT using wireless technologies.

## 6. Conclusions

In conclusion, in our group of UHL patients we found a significant improvement in self-reported auditory abilities and speech-in-noise perception when HA fitting was followed by wireless AT as opposed to conventional AT. Wireless AT facilitated habituation to HAs and binaural hearing better than conventional AT. Our results should be confirmed by larger multicenter studies. Our findings suggest that future interventions might benefit from incorporating wireless technology in AT programs.

## Figures and Tables

**Table 1:** Unilateral hearing loss etiology according to group distribution.

Hearing Loss Etiology	Patients in Study Group	Patients in Control Group	Chi-Squared
Sudden sensorineural hearing loss	16	20	statistic: 0.444
			*p*-value: 0.505
Trauma	8	11	statistic: 0.474
			*p*-value: 0.491
Chronic otitis media	8	6	statistic: 0.286
			*p*-value: 0.593
Otosclerosis	4	3	statistic: 0.143
			*p*-value: 0.705
Unknown	10	10	statistic: 0.0
			*p*-value: 1.0
Total	46	50	

**Table 2:** Comparison of demographics and clinical characteristics between the two groups without hearing aids.

	Study Group	Control Group	*p*-Value *
Female/Male	20/26	26/24	0.53
Age (years)	56.7 ± 8.5	58.2 ± 9	0.9
Tinnitus (yes/no)	28/18	34/16	0.6
Mean duration of hearing loss (years)	10.2 ± 3.2	9.4 ± 3.0	0.81
Mean pure tone average (dB)	59.1 ± 8	56.2 ± 7.5	0.43
Mean speech-in-noise with SNR 0 (%)	15 ± 5%	16 ± 5%	0.91
Mean speech-in-noise with SNR +5 (%)	49 ± 6%	50 ± 6%	0.93
Mean speech-in-noise with SNR +10 (%)	76 ± 4%	76 ± 4%	1.0

* = Mann–Whitney U test or chi-square test as appropriate. Abbreviations: SNR = signal-to-noise ratio.

**Table 3:** Speech-in-noise results of the two groups with different signal-to-noise ratios (SNRs) before and after hearing aid (HA) fitting and auditory training (AT).

	Before HA Fitting and AT	After HA Fitting and AT	*p*-Value *
*Study group*			
Mean speech-in-noise SNR 0	15 ± 4%	33 ± 5%	0.12
Mean speech-in-noise SNR +5	50 ± 6%	85 ± 4%	0.002
Mean speech-in-noise SNR +10	76 ± 3%	98 ± 2%	0.02
Mean total SSQ	4.1 ± 1.1	7.3 ± 0.6	0.03
*Control group*			
Mean speech-in-noise SNR 0	16 ± 4%	26 ± 5%	0.39
Mean speech-in-noise SNR +5	49 ± 6%	65 ± 5%	0.16
Mean speech-in-noise SNR +10	76 ± 3%	90 ± 4%	0.08
Mean total SSQ	4.8 ± 2.4	7.0 ± 2.5	0.7

* Wilcoxon signed-rank and Mann–Whitney U test.

## Data Availability

Data can be provided from the corresponding authors upon reasonable request.
